# Aging metrics incorporating cognitive and physical function capture mortality risk: results from two prospective cohort studies

**DOI:** 10.1186/s12877-022-02913-y

**Published:** 2022-04-28

**Authors:** Xingqi Cao, Chen Chen, Jingyun Zhang, Qian-Li Xue, Emiel O. Hoogendijk, Xiaoting Liu, Shujuan Li, Xiaofeng Wang, Yimin Zhu, Zuyun Liu

**Affiliations:** 1 Center for Clinical Big Data and Analytics of the Second Affiliated Hospital and Department of Big Data in Health Science School of Public Health, Zhejiang University School of Medicine, Zhejiang, Hangzhou China; 2 China CDC Key Laboratory of Environment and Population Health, National Institute of Environmental Health; National Center for AIDS/STD Control and Prevention, Chinese Center for Disease Control and Prevention, Beijing, China; 3 Department of Medicine Division of Geriatric Medicine and Gerontology and Center on Aging and Health, Johns Hopkins Medical Institutions, Baltimore, MD USA; 4 Department of Epidemiology and Data Science, Amsterdam Public Health research institute, Amsterdam UMC – location VU University Medical Center, Amsterdam, the Netherlands; 5 School of Public Affairs, Zhejiang University, Zhejiang, Hangzhou China; 6 Department of Neurology, Beijing Chaoyang Hospital, Capital Medical University, Beijing, China; 7 National Clinical Research Center for Aging and Medicine, Huashan Hospital, and Human Phenome Institute, Fudan University, Shanghai, China; 8 Department of Epidemiology and Biostatistics, School of Public Health, Zhejiang University School of Medicine, Zhejiang, Hangzhou China

**Keywords:** Cognitive frailty, Cognitive impairment, Frailty index, Motoric cognitive risk syndrome, Physical frailty

## Abstract

**Background:**

Aging metrics incorporating cognitive and physical function are not fully understood, hampering their utility in research and clinical practice. This study aimed to determine the proportions of vulnerable persons identified by three existing aging metrics that incorporate cognitive and physical function and the associations of the three metrics with mortality.

**Methods:**

We considered three existing aging metrics including the combined presence of cognitive impairment and physical frailty (CI-PF), the frailty index (FI), and the motoric cognitive risk syndrome (MCR). We operationalized them using data from the China Health and Retirement Longitudinal Study (CHARLS) and the US National Health and Nutrition Examination Survey (NHANES). Logistic regression models or Cox proportional hazards regression models, and receiver operating characteristic curves were used to examine the associations of the three metrics with mortality.

**Results:**

In CHARLS, the proportions of vulnerable persons identified by CI-PF, FI, and MCR were 2.2, 16.6, and 19.6%, respectively. Each metric predicted mortality after adjustment for age and sex, with some variations in the strength of the associations (CI-PF, odds ratio (OR) (95% confidence interval (CI)) 2.87 (1.74–4.74); FI, OR (95% CI) 1.94 (1.50–2.50); MCR, OR (95% CI) 1.27 (1.00–1.62)). CI-PF and FI had additional predictive utility beyond age and sex, as demonstrated by integrated discrimination improvement and continuous net reclassification improvement (all *P* < 0.001). These results were replicated in NHANES.

**Conclusions:**

Despite the inherent differences in the aging metrics incorporating cognitive and physical function, they consistently capture mortality risk. The findings support the incorporation of cognitive and physical function for risk stratification in both Chinese and US persons, but call for caution when applying them in specific study settings.

**Supplementary Information:**

The online version contains supplementary material available at 10.1186/s12877-022-02913-y.

## Background

The aging process is characterized by deteriorations across a broad spectrum of physiological systems over time. To quantify the complex aging process, aging metrics have been developed in molecular, phenotypic, and functional domains [1]. Functional metrics of aging include cognitive and physical function. It has been observed that age-related declines in cognitive and physical function coexist in many older persons, implying possible shared mechanisms underlying the two functional aspects [2]. Furthermore, older persons have increased risk of poor prognosis (e.g., disability [3, 4], death [4–8]) when having problems in cognitive and physical function simultaneously. The potential link between cognitive and physical function motivate many researchers to explore aging metrics that incorporate the two functional aspects [9–11]. Such composite aging metrics could serve as a new target for preventing or delaying the onset of disability and extending healthy life expectancy in older persons [12, 13].

To date, there are three main aging metrics reported in the literature (Fig. S1A in the Additional file 1, see details in [14]). First, in 2013, an International Association of Gerontology and Geriatrics consensus group proposed cognitive frailty as the simultaneous presence of both cognitive impairment and physical frailty (PF) in non-demented older persons [15] (referred to as CI-PF). PF represents a state of increased vulnerability to stressor resulting from cumulative declines in multiple physiological systems [16, 17]. Second, the frailty index (FI) integrates deficits across multiple domains including cognitive and physical function, resulting in a score reflecting risks across various outcomes (e.g., hospitalization and death) [18]. Finally, Verghese et al. [19] proposed the motoric cognitive risk syndrome (MCR), characterized by the simultaneous presence of subjective cognitive complaints and slow gait. The latter has been widely used to operationalize physical function [20]. Despite some conceptual overlap in the three metrics above, there are substantial differences in their operationalizations and characteristics, which have not been formally evaluated, hampering their utility in research and clinical practice.

In this study, we performed comprehensive analyses to describe the three metrics using data from two national prospective cohort studies: the China Health and Retirement Longitudinal Study (CHARLS) and the US National Health and Nutrition Examination Survey (NHANES). We first described the proportions of persons identified as vulnerable by the three aging metrics (CI-PF, FI, and MCR) in the same population. Next, we examined the associations of the three metrics with mortality.

## Methods

### Study population

The CHARLS was approved by the Biomedical Ethics Review Committee of Peking University, and all persons provided informed consent. As shown in Fig. S1B in the Additional file 1, out of 17,708 persons aged 45 years and older enrolled in the baseline survey (2011/2012), we excluded those aged below 60 years (*N* = 10,255; because gait speed was measured only in persons aged 60 years and over), with missing data on covariates (*N* = 6), missing data on gait speed (*N* = 1953), who had disability in basic activities of daily living (BADL) (*N* = 1462), or had the memory-related disease (e.g., Alzheimer’s disease, brain atrophy, and Parkinson’s disease) (*N* = 103), leaving the analytic sample 1 of 3929 persons aged 60–95 years.

Persons in NHANES were first recruited in the 1999–2002 cycle. The NHANES was approved by the National Center for Health Statistics Research Ethics Review Board, and all persons provided informed consent. Out of the 9882 persons aged 20 years and older, we excluded persons with missing data on gait speed (*N* = 5352; the walk testing was only measured in participants who aged 50 years and older. Gait speed measurement was needed to construct both PF and MCR. But, from 2003 to 2004 cycle on, gait speed was no longer captured in NHANES), final mortality status (*N* = 3), who had disability in BADL (*N* = 510), or had dementia (*N* = 167), leaving the analytic sample 2 of 3850 persons aged 50–85 years.

A more detailed description of the study population is provided in Additional file 2.

### Measures

#### Aging metrics incorporating cognitive and physical function

As mentioned above, we considered three metrics incorporating cognitive and physical function: CI-PF, FI, and MCR (see details in the Additional file 2).

#### CI-PF

The CI-PF was defined as the simultaneous presence of both cognitive impairment and PF in non-demented older persons, proposed in 2013 by an international consensus group) [15]. PF was measured using the Fried frailty phenotype approach [17], and had been previously developed and validated in the CHARLS [21] and NHANES [22], respectively. Persons were classified as frail if they met ≥3 of the five items; otherwise, they were classified as non-frail. Based on the two components, i.e., cognitive impairment and PF, we defined four combined groups as done in previous studies [11]: normal cognition & non-frailty, cognitive impairment & non-frailty, normal cognition & frailty, and cognitive impairment & frailty. The cognitive impairment & frailty group was defined as vulnerable.

#### Fi

The FI was based on the degree of accumulation of health deficits and represented an alternative instrument of frailty that incorporates many health dimensions (e.g., comorbidities and disabilities) including cognition [18]. The FI was calculated as a ratio of the number of deficits in a person out of the total possible deficits considered [23], with a range of 0 to 1. A FI ≤0.10 was considered as non-frail, 0.10 < FI ≤ 0.21 was pre-frail, and FI > 0.21 was frail [24]. The group with frailty was defined as vulnerable.

#### MCR

The MCR was defined as the simultaneous presence of both subjective cognitive complaints and objective slow gait, in the absence of a diagnosis of dementia and disability in BADL [25]. The group of persons with MCR was defined as vulnerable.

#### Mortality

The death information in CHARLS was collected from the exit interview in 2013, 2015, and 2018 waves. Because the exact date of death was not available in the 2015 and 2018 waves, we constructed a binary variable to denote the occurrence of death within the 6-year follow-up since baseline in this study. Thus, we excluded participants without death information (i.e., those lost to follow-up) in CHARLS in this study. All-cause mortality during approximately 13.8-year follow-up in NHANES was based on linked data from records taken from the National Death Index through December 31, 2015.

#### Covariates

Covariates in CHARLS including age, sex, residence, education, and chronic disease were collected at baseline. The residence was defined as urban or rural. Education level was defined as illiterate, elementary school, middle school, high school, or college and higher than college. Chronic diseases included 10 self-reported conditions: hypertension, diabetes or high blood sugar, cancer or malignant tumor, chronic lung disease, heart problems, stroke, kidney disease, stomach or other digestive diseases, arthritis or rheumatism, and asthma. The total number of chronic diseases was calculated. We classified disease counts into five categories: 0 disease, 1 disease, 2 diseases, 3 diseases, and 4 or more diseases.

Covariates in NHANES including age, sex, education, race/ethnicity, and chronic disease were collected at baseline. Education level was defined as less than high school (HS), HS/general educational development (GED), some college (having attended college but not receiving at least a bachelor’s degree), or college (having a bachelor’s degree or higher). Racial/ethnic group was defined as non-Hispanic white, non-Hispanic black, or Hispanic. Chronic diseases included 10 self-reported conditions: congestive heart failure, stroke, cancer, chronic bronchitis, emphysema, cataracts, arthritis, type 2 diabetes, hypertension, and myocardial infarction. We classified disease counts into five categories as done in CHARLS.

### Statistical analysis

The analytic plan for this study was described in Fig. S1B in the Additional file 1. In analysis 1, we first described the characteristics of the full sample of CHARLS and NHANES, as well as the characteristics of vulnerable persons defined by the three metrics using mean (± standard deviation [SD]) or counts (percentages). We then plotted the proportions of vulnerable persons identified by the three aging metrics at baseline in the full sample, as well as stratified by age categories (< 65 years, and ≥ 65 years) and sex. To check the consistency of the three metrics, we further presented the distribution of persons with CI-PF and MCR across the FI groups.

In analysis 2, we evaluated the associations of the three metrics with all-cause mortality. We used logistic regression models in CHARLS (because the exact timing of death during the follow-up period was unknown in CHARLS) and Cox proportional hazards regression models in NHANES. Model 1 adjusted for age and sex. Model 2 additionally adjusted for education, and residence (CHARLS) or ethnicity/race (NHANES). We considered these covariates as they may confound the associations of the three metrics with mortality. For logistic regression models, we documented odds ratios (ORs) and corresponding 95% confidence intervals (CIs). For Cox proportional hazards regression model, we documented hazard ratios (HRs) and corresponding 95% CIs. Then, receiver operating characteristic (ROC) curves were used to evaluate the utility of the three metrics for mortality prediction beyond basic models with age and sex, in both CHARLS and NHANES. ROC curves were plotted using the R package “pROC”. Indices including the delta C-statistic, integrated discrimination improvement (IDI), and continuous net reclassification improvement (NRI) were calculated, in comparison to that of the basic models. Delta C-statistic equals to x% means that the difference in predicted risks between the persons with and without the outcome increased by x% in the updated models. IDI equals to x% means that the difference in average predicted risks between the persons with and without the outcome increased by x% in the updated model. Continuous NRI equals to x% means that compared with persons without outcome, persons with outcome were almost x% more likely to move up a category than down. IDI and continuous NRI were calculated using the R package “PredictABEL”.

Analyses were performed using R version 3.6.3 (2020-02-29) and SAS version 9.4 (SAS Institute, Cary, NC). A *P* value of < 0.05 (two-tailed) was considered statistically significant.

## Results

### The characteristics of the study population

As shown in Table 1, the mean ages (SD) of the 3929 persons in CHARLS and the 3850 persons in NHANES were 67.4 (SD = 6.3) years and 65.6 (SD = 9.6) years, respectively. The proportions of males were 53.5% in CHARLS and 50.1% in NHANES. There were also differences in characteristics among the three groups defined as vulnerable. For example, in CHARLS, the mean age of persons was 73.2 (SD = 7.8) years for those defined as vulnerable according to CI-PF, for the FI this value was 68.7 (SD = 7.1), and for MCR this value was 66.0 (SD = 5.4). In all vulnerable groups, proportions of males (32.9% for CI-PF, 41.2% for FI, and 41.4% for MCR) were lower than that of females. The characteristics of cognition status by age groups in CHARLS 2011/2012 and NHANES 1999–2002 are presented in Table S1A in the Additional file 3. Differences in characteristics were found between excluded and included populations. Those who were included were more likely to be older and males (Table S1B in the Additional file 3).

**Table 1:** Summary characteristics of the total sample and for vulnerable persons identified by the three aging metrics, CHARLS 2011/2012 and NHANES 1999–2002

Characteristics	CHARLS				NHANES			
	Total	Vulnerable persons			Total	Vulnerable persons		
		CI-PF	FI	MCR		CI-PF	FI	MCR
N	3929	85	653	770	3850	104	1035	167
Age, mean ± SD	67.4 ± 6.3	73.2 ± 7.8	68.7 ± 7.1	66.0 ± 5.4	65.6 ± 9.6	72.0 ± 7.3	69.6 ± 9.3	71.6 ± 9.4
Male, %	2102 (53.5)	28 (32.9)	269 (41.2)	319 (41.4)	1927 (50.1)	46 (44.2)	468 (45.2)	83 (49.7)
Residence, rural, %	2427 (61.8)	65 (76.5)	435 (66.6)	533 (69.2)	─	─	─	─
Race/Ethnicity ^a^, %								
Non-Hispanic white	─	─	─	─	2130 (56.6)	47 (46.1)	545 (53.6)	84 (51.5)
Non-Hispanic black	─	─	─	─	673 (17.9)	20 (19.6)	194 (19.1)	23 (14.1)
Hispanic	─	─	─	─	957 (25.5)	35 (34.3)	278 (27.3)	56 (34.4)
Education ^b^, %								
Category 1	1296 (33.0)	61 (71.8)	269 (41.2)	334 (43.4)	1496 (39.0)	71 (68.3)	526 (51.0)	90 (54.9)
Category 2	1859 (47.3)	24 (28.2)	297 (45.5)	352 (45.7)	868 (22.6)	17 (16.3)	213 (20.7)	23 (14.0)
Category 3	511 (13.0)	0	60 (9.2)	71 (9.2)	799 (20.8)	8 (7.7)	184 (17.8)	28 (17.1)
Category 4	195 (5.0)	0	19 (2.9)	11 (1.4)	677 (17.6)	8 (7.7)	108 (10.5)	23 (14.0)
Category 5	68 (1.7)	0	8 (1.2)	2 (0.3)	─	─	─	─
Disease counts ^c^, %								
0	1116 (28.4)	23 (27.1)	40 (6.0)	173 (22.5)	805 (20.9)	1 (1.0)	14 (1.4)	12 (7.2)
1	1252 (31.9)	30 (35.3)	107 (16.0)	252 (32.7)	1138 (29.6)	17 (16.3)	126 (12.2)	20 (12.0)
2	885 (22.5)	17 (20.0)	166 (24.8)	178 (23.1)	961 (25.0)	19 (18.3)	244 (23.6)	47 (28.1)
3	426 (10.8)	12 (14.1)	169 (25.3)	100 (13.0)	574 (14.9)	28 (26.9)	327 (31.6)	37 (22.2)
≥ 4	250 (6.4)	3 (3.5)	187 (28.0)	67 (8.7)	372 (9.7)	39 (37.5)	324 (31.3)	51 (30.5)
Cognitive impairment, %	1348 (34.3)	/	307 (47.0)	397 (51.6)	1055 (27.4)	/	411 (39.7)	70 (41.9)
Physical frailty, %	165 (4.2)	/	78 (11.9)	78 (10.1)	338 (8.8)	/	302 (29.2)	73 (43.7)
Gait speed, mean ± SD	0.7 ± 0.3	0.4 ± 0.3	0.6 ± 0.3	0.5 ± 0.1	1.0 ± 0.3	0.6 ± 0.2	0.8 ± 0.2	0.8 ± 0.3
Slow gait, %	1979 (50.4)	70 (82.4)	396 (60.6)	/	1252 (32.5)	71 (68.3)	449 (43.4)	/
Cognitive complaints, %	1428 (36.4)	54 (63.5)	381 (58.4)	/	409 (10.6)	28 (26.9)	289 (27.9)	/
The cognition score ^d^, mean ± SD	9.8 ± 4.3	3.9 ± 2.2	8.3 ± 4.3	8.0 ± 4.1	42.0 ± 14.7	23.2 ± 10.7	37.9 ± 14.3	35.5 ± 12.8

*CHARLS* China Health and Retirement Longitudinal Study, *NHANES* National Health and Nutrition Examination Survey, *CI-PF* cognitive impairment and physical frailty, *FI* frailty index, *MCR* Motoric Cognitive Risk syndrome, *SD* standard deviation. CI-PF, FI, and MCR indicate vulnerable persons identified by the three aging metrics, as described in Methods

*Notes*: ^a^Ninety persons who self-identified as other races (including multi-racial) were excluded

^b^Ten persons with missing data on education were excluded. In CHARLS, category 1 to 5 indicates “illiteracy”, “elementary”, “middle”, “senior” and “college and higher than college”, respectively; In NHANES, category 1 to 4 indicates “lower than high school”, “high school or general educational development”, “some college”, and “college”, respectively

^c^In CHARLS, chronic diseases included hypertension, diabetes or high blood sugar, cancer or malignant tumor, chronic lung disease, heart problems, stroke, kidney disease, stomach or other digestive diseases, arthritis or rheumatism, and asthma. In NHANES, chronic diseases included congestive heart failure, stroke, cancer, chronic bronchitis, emphysema, cataracts, arthritis, type 2 diabetes, hypertension, and myocardial infarction

^d^In CHARLS, cognitive function was assessed by three tests, including the Telephone Interview of Cognitive Status-10 (TICS-10), word recall, and figure drawing. In NHANES, cognitive function was assessed by the Digit Symbol Substitution Test (DSST)

### How many persons are identified as vulnerable by the three metrics combining cognitive and physical function?

**Fig. 1:**
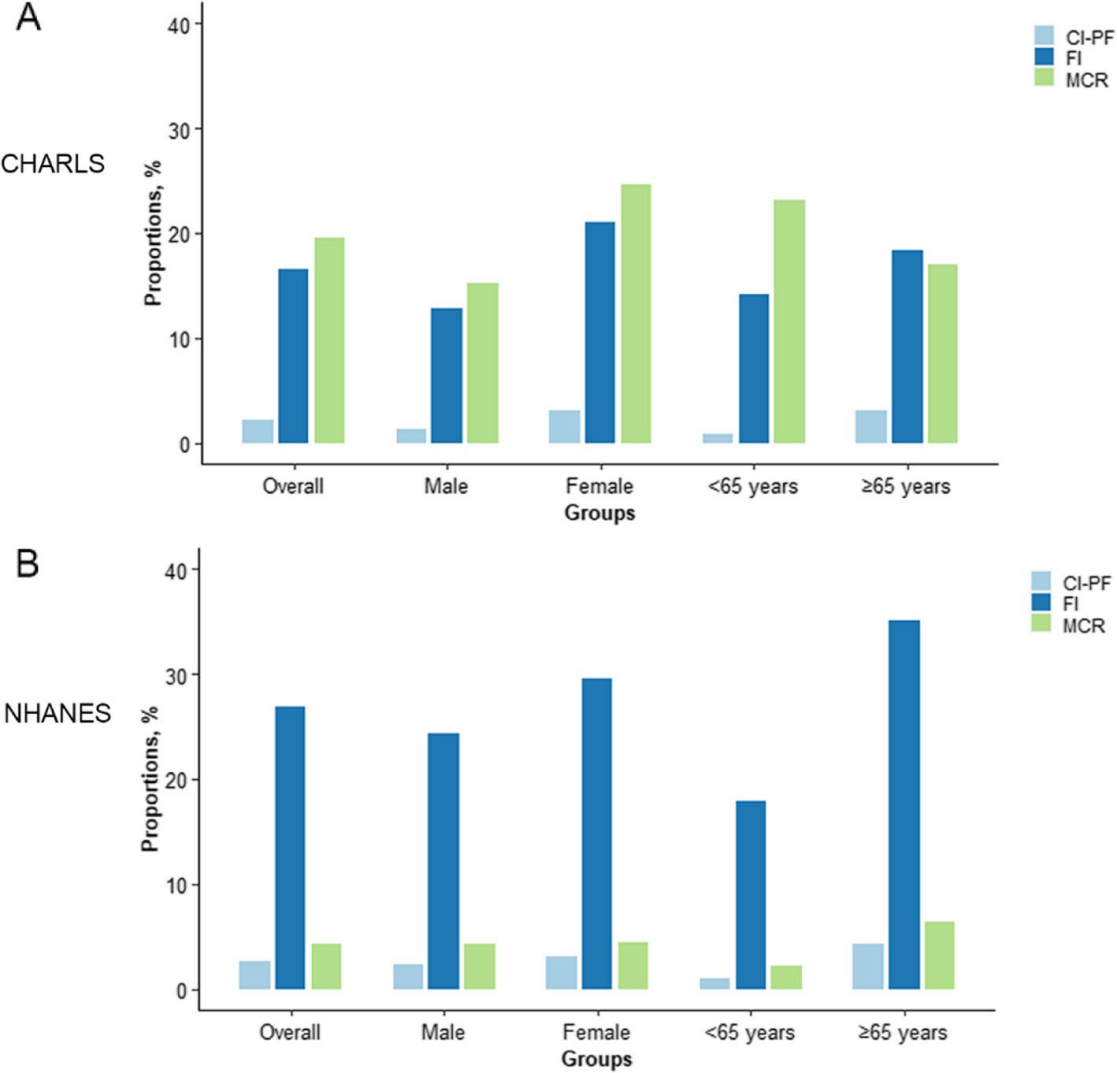
Proportions of vulnerable persons identified by the three aging metrics incorporating cognitive and physical function in CHARLS (**A**) and NHANES (**B**). CHARLS, China Health and Retirement Longitudinal Study; NHANES, National Health and Nutrition Examination Survey; CI-PF, cognitive impairment and physical frailty; FI, frailty index; MCR, Motoric Cognitive Risk syndrome

As shown in Fig. 1, we observed large variations in the proportions of vulnerable persons using the three metrics in CHARLS and NHANES. In CHARLS, the proportions of vulnerable persons identified by CI-PF, FI, and MCR were 2.2, 16.6, and 19.6%, respectively (Table S1C in the Additional file 3 and Fig. 1). In NHANES, the proportions of vulnerable persons identified by CI-PF, FI, and MCR were 2.7, 26.9, and 4.3%, respectively. We further presented the distribution of persons with CI-PF and MCR across the FI groups in CHARLS and NHANES. We found that in CHARLS, persons who were non-frail and pre-frail defined by FI, 26.2 and 36.2% belonged to the cognitive impairment & non-frailty group for CI-PF, and 10.6 and 23.3% were classified as MCR.

### Do aging metrics incorporating cognitive and physical function predict mortality?

Table 2 presents the associations of the three metrics (CI-PF, FI, and MCR) with all-cause mortality. When using the CI-PF, compared with the normal cognition & non-frailty group, the multivariable-adjusted ORs (95% CIs) or HRs (95% CIs) of the cognitive impairment & non-frailty group, normal cognition & frailty group, and cognitive impairment & frailty group for all-cause mortality were 1.35 (1.08–1.69), 1.69 (0.99–2.90), and 2.42 (1.46–4.02) in CHARLS (as previously reported [11]), and 1.39 (1.23–1.57), 3.09 (2.58–3.69), and 2.78 (2.19–3.54) in NHANES, respectively. When using the FI, compared with the non-frail group, the multivariable-adjusted ORs (95% CI) or HRs (95% CI) of the pre-frail group and the frail group were 1.27 (1.02–1.57), and 1.82 (1.41–2.35) in CHARLS, and 1.36 (1.18–1.57), and 2.58 (2.23–2.98) in NHANES, respectively. When using the MCR, compared with persons without MCR, the multivariable-adjusted OR (95% CI) or HR (95% CI) of persons with MCR were 1.16 (0.91–1.47) in CHARLS, and 1.83 (1.51–2.22) in NHANES, respectively. Further adjustment for number of comorbidities did not substantially change the strength and significance of the above associations (Table S2 in the Additional file 4). Moreover, we found that vulnerable persons, as defined by the three aging metrics, had a much steeper decline in survival over approximately 13.8 years of follow-up in NHANES (Fig. S2 in the Additional file 4).

**Table 2:** Associations of the three aging metrics incorporating cognitive and physical function with all-cause mortality

Aging metrics		Model 1	Model 2
**CHARLS**		**OR (95% CI)**	**OR (95% CI)**
**CI-PF**^**a**^	Normal cognition & non-frailty	Ref	Ref
	Cognitive impairment & non-frailty	1.59 (1.29, 1.96)	1.35 (1.08, 1.69)
	Normal cognition & frailty	1.88 (1.10, 3.20)	1.69 (0.99, 2.90)
	Cognitive impairment & frailty	2.87 (1.74, 4.74)	2.42 (1.46, 4.02)
**FI**	Non-frail	Ref	Ref
	Pre-frail	1.34 (1.08, 1.66)	1.27 (1.02, 1.57)
	Frail	1.94 (1.50, 2.50)	1.82 (1.41, 2.35)
**MCR**	Absence	Ref	Ref
	Presence	1.27 (1.00, 1.62)	1.16 (0.91, 1.47)
**NHANES**		**HR (95% CI)**	**HR (95% CI)**
**CI-PF**	Normal cognition & non-frailty	Ref	Ref
	Cognitive impairment & non-frailty	1.39 (1.25, 1.56)	1.39 (1.23, 1.57)
	Normal cognition & frailty	3.16 (2.66, 3.76)	3.09 (2.58, 3.69)
	Cognitive impairment & frailty	2.85 (2.26, 3.60)	2.78 (2.19, 3.54)
**FI**	Non-frail	Ref	Ref
	Pre-frail	1.39 (1.21, 1.60)	1.36 (1.18, 1.57)
	Frail	2.68 (2.33, 3.10)	2.58 (2.23, 2.98)
**MCR**	Absence	Ref	Ref
	Presence	1.91 (1.58, 2.31)	1.83 (1.51, 2.22)

*CHARLS* China Health and Retirement Longitudinal Study, *OR* odds ratio, *CI* confidence interval, *CI-PF* cognitive impairment and physical frailty, *FI* frailty index, *MCR* Motoric Cognitive Risk syndrome, *NHANES* National Health and Nutrition Examination Survey, *HR* hazard ratio

*Notes*: ^a^As previously reported (Chen et al., 2020), significant differences in all-cause mortality between the four CI-PF groups were observed

Model 1: adjusted for age, and sex

Model 2: adjusted for age, sex, education, and residence (in CHARLS) or ethnicity/race (in NHANES)

**Fig. 2:**
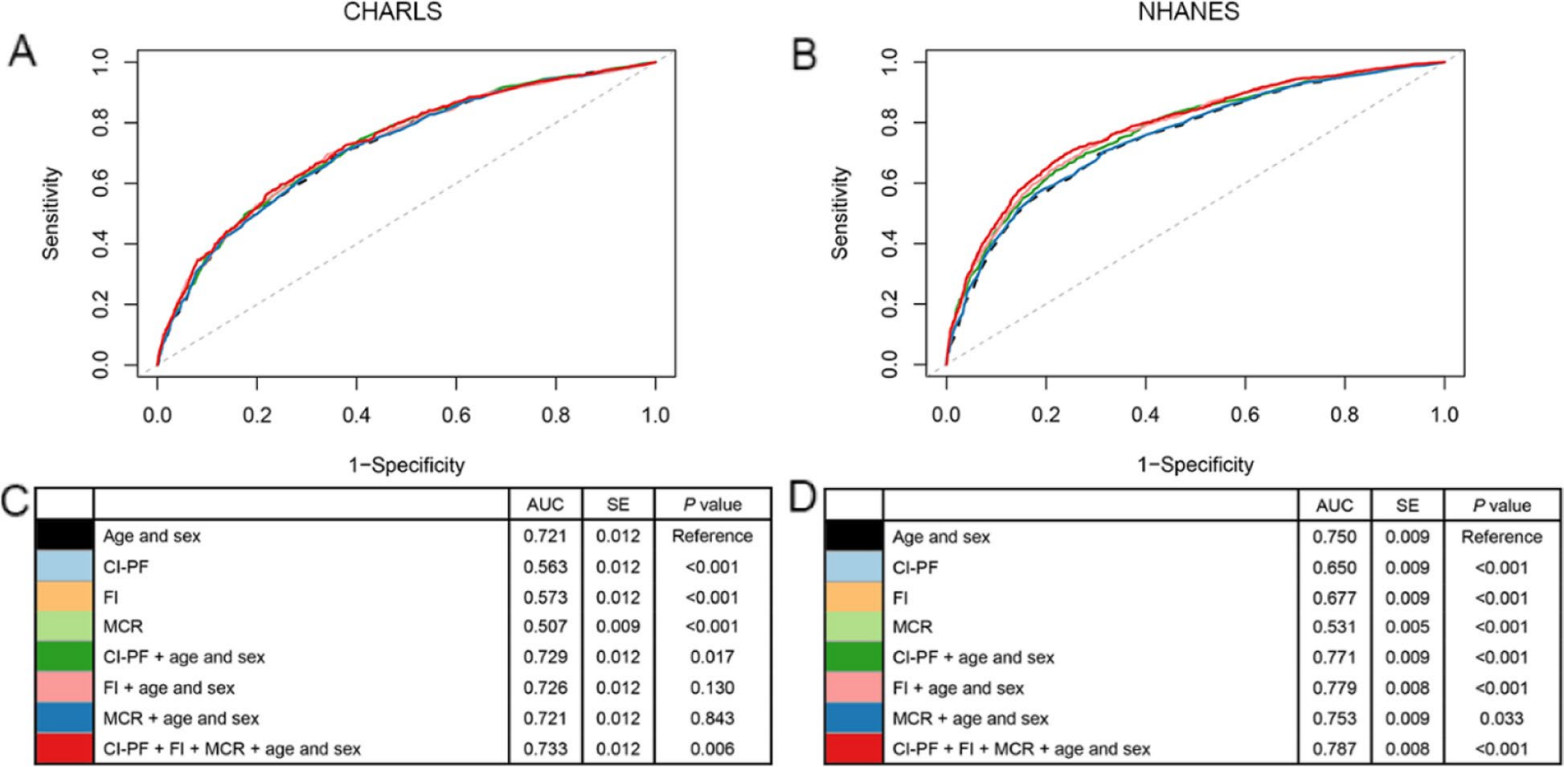
Receiver-operator characteristics (ROC) curves for prediction of all-cause mortality in CHARLS and NHANES. CHARLS, China Health and Retirement Longitudinal Study; NHANES, National Health and Nutrition Examination Survey; CI-PF, cognitive impairment and physical frailty; FI, frailty index; MCR, Motoric Cognitive Risk syndrome; AUC, area under the curve; SE, standard error. A and B show ROC curves for the prediction of all-cause mortality. C and D show the AUC for each model. A and C are based on the CHARLS. B and D are based on the NHANES

As shown in Fig. 2, we examined the ROC curves using various models, such as the basic model with age and sex only, and the basic model with or without the three metrics included. We found that in almost all cases, the three metrics added predictive utility, except for MCR in CHARLS. The results suggested that aging metrics incorporating cognitive and physical function capture something above and beyond what can be explained by age and sex when predicting mortality. Compared with the basic model (with age and sex only), the models including CI-PF or FI (only in NHANES) had better discrimination ability, as demonstrated by significantly increased C-statistics (0.008–0.029, Table 3). The better performance of CI-PF and FI was further demonstrated by significant improvements in reclassification as assessed by IDI (range: 0.009–0.043) and continuous NRI (range: 0.155–0.568).

**Table 3:** The reclassification performance and improvement in discrimination by the three aging metrics incorporating cognitive and physical function^a^

Aging metrics	Delta C-statistic	***P*** value	IDI	***P*** value	NRI	***P*** value
**CHARLS**						
CI-PF	0.008	0.017	0.011	< 0.001	0.211	< 0.001
FI	0.006	0.130	0.009	< 0.001	0.155	< 0.001
MCR	0.0003	0.843	0.001	0.035	−0.027	0.441
**NHANES**						
CI-PF	0.021	< 0.001	0.033	< 0.001	0.568	< 0.001
FI	0.029	< 0.001	0.043	< 0.001	0.332	< 0.001
MCR	0.003	0.033	0.007	< 0.001	0.125	< 0.001

*IDI* integrated discrimination improvement, *NRI* net reclassification index, *CHARLS* China Health and Retirement Longitudinal Study, *CI-PF* cognitive impairment and physical frailty, *FI* frailty index, *MCR* Motoric Cognitive Risk syndrome, *NHANES* National Health and Nutrition Examination Survey

*Notes*: ^a^We calculated the IDI and continuous NRI using the R package “PredictABEL”, in comparison to that of the model with age and sex

## Discussion

Aging metrics incorporating cognitive and physical function are an emerging topic in gerontological and geriatric research, particularly due to its high predictive ability of poor prognosis. Various metrics have been developed, such as the three metrics mentioned in this study: CI-PF, FI, and MCR. There is a lack of understanding of how these metrics work when applying them in the same population in terms of identifying vulnerable persons and capturing the risk of adverse health outcomes. We showed that there was variability in the three metrics concerning the proportion of persons defined as vulnerable and we provided a full picture showing how the three metrics captured mortality risk in two large national cohorts (CHARLS and NHANES).

First, we noted the differences in the proportions of vulnerable persons identified by the three metrics. In this study, the proportions of vulnerable persons defined by the CI-PF in CHARLS and NHANES were within that from previous studies, ranging from 1.0 to 12.1% among the community-dwelling persons [3, 11, 26]. However, the proportion of vulnerable persons defined by the FI was relatively higher than that of the CI-PF measurement. The great variability in the proportions of vulnerable persons is not surprising and may be due to many factors [27–30], such as the age ranges and sex proportions of the sample, and the different setting of studies. Second, the differences mentioned above imply that the three metrics may capture varying dimensions of functional aging. For instance, in CHARLS persons who were non-frail defined by FI, 26.2% were classified as cognitive impairment & non-frailty group for CI-PF, and 10.6% were classified as MCR. This is because cognitive function is not heavily weighted in FI although it is one of the many domains included in the FI measurement. The heterogeneity we observed confirms that different metrics combining cognitive and physical function cannot be assumed to be interchangeable.

Consistent with previous studies, we found that the three metrics capture mortality risk [4]. But, CI-PF and FI present stronger predictive utility of mortality than MCR in both Chinese and US persons despite the relatively low effect sizes. This has not been reported previously. The PF, included in the CI-PF, is an objective metric (e.g., grip strength and gait speed), and is widely used in research because of its ability to predict adverse health outcomes [31]. The better performance of the FI in predicting mortality risk could be attributed to the multidimensional and accumulative nature of the deficit approach, which could capture differences in the health status of persons at the same age [18]. The MCR is not a strong predictor of mortality relative to the other two metrics, intuitively because of its requirement to exclude persons with BADL disability. Theoretically, the MCR was designed to capture early signals of cognitive decline or functional changes occurring many years before the end of life.

However, CI-PF and FI may be inadvisable metrics in clinical settings. The CI-PF requires objective measures of cognitive impairment and PF, demanding much time and efforts [32]. The multidimensional nature of the FI measurement impedes its feasibility in large population studies. In addition to including sufficient numbers of deficits (at least 30), indeed, various FI scores differ significantly concerning their complexity as well as the stability in terms of the predictions of adverse outcomes [33]. Thus, we suggest that the feasibility and performance of the three metrics should be carefully balanced when using them.

This study has important implications. First, despite the heterogeneity of the three metrics, consistent associations with mortality were observed, supporting that cognitive and physical function have something in common, such as pathological mechanisms [34, 35]. This provides us with the opportunity to better track the future health trajectories of frail persons with cognitive impairment. Second, the predictive utility of these aging metrics including CI-PF and FI supports the implementation of targeted interventions and health education at an early stage, which could effectively reduce mortality risk at a lower cost. With appropriate managements, it is expected to alleviate the burden of health care in those with varying cognitive and physical status.

Our study has several strengths. First, the two cohorts included in our study are representative samples from two of the largest countries (China and US) in the world, which substantially differ in many aspects such as social-economic position and lifestyle. The consistent results from the two cohort studies strengthened our findings. Second, we presented the three aging metrics in the same population and examine their associations with mortality, which is scarce in the literature. Nevertheless, several limitations of this study should be mentioned. First, the CHARLS has a relatively short follow-up period, impeding us to evaluate the long-term effect of cognitive and physical impairment on adverse health outcomes in this cohort. Second, exclusion of 5352 participants in NHANES 1999–2002 who did not have data on gait speed may result in that the last two subgroups of CI-PF were not representative of their original whole populations. Third, the items included in FI were mostly related to comorbidities, BADL, and instrumental activities of daily living, and only one item was related to cognition; thus, we suggest that it’s difficult to justify that the FI presents cognitive impairment risk. Fourth, it should be noted that there are differences between study samples of CHARLS and NHANES, such as ethnic differences and age ranges. These may lead to variation in results between the two samples. However, we demonstrated the similar associations of the three aging metrics incorporating cognitive and physical function and mortality in the two different populations. Finally, there were differences in the components of the three metrics across the two cohorts. Nevertheless, the metrics have been proved to be valid in previous studies [22, 32, 36]. Also, the three metrics are different in terms of target population and initial purpose. Thus, applying them in different settings should be done with caution.

## Conclusions

In both Chinese and US persons, we found that aging metrics incorporating cognitive and physical function consistently capture mortality risk, despite their inherent substantial differences. The incorporation of cognitive and physical function has the potential for risk stratification in both research and clinical settings. The findings support the implementation of preventive strategies and intervention programs targeting these metrics to improve the quality of life and further reduce premature death in aging societies.

## Supplementary Information

**Additional file 1: Figure S1.** Roadmap for the comprehensive analyses of the three aging metrics incorporating cognitive and physical function.

**Additional file 2.** Detailed description of the Methods.

**Additional file 3: Table S1A.** Summary characteristics of the cognition status among different age groups, CHARLS 2011/2012 and NHANES 1999–2002. **Table S1B.** Characteristics of the included and excluded population. **Table S1C.** Distribution of the participants with CI-PF and MCR across the FI groups, CHARLS 2011/2012 and NHANES 1999–2002.

**Additional file 4: Table S3.** Associations of the three aging metrics incorporating cognitive and physical function with all-cause mortality when additionally adjusting for comorbidities. **Figure S2.** Kaplan-Meier survival curves of the groups defined by the three aging metrics in NHANES.

**Additional file 5: **Sensitivity Analyses Results. **Table S4.** Associations of the three aging metrics incorporating cognitive and physical function with all-cause mortality among older adults aged 60 years and over in NHANES (*N* = 2751). **Figure S3.** Association of the three aging metrics with all-cause mortality among older adults aged 60 years and over in NHANES (*N* = 2751).

## Data Availability

The datasets generated and analyzed during the current study are available in the CHARLS and NHANES website, available in http://charls.pku.edu.cn/en and https://www.cdc.gov/nchs/nhanes/index.htm, respectively.

## Abbreviations

BADL: Basic activities of daily living
AUC: Area under the curve
CHARLS: The China Health and Retirement Longitudinal Study
CI-PF: Cognitive impairment and physical frailty
CI: Confidence interval
DSST: The Digit Symbol Substitution Test
FI: Frailty index
HR: Hazard ratio
IDI: Integrated discrimination improvement
MCR: Motoric cognitive risk syndrome
NHANES: The US National Health and Nutrition Examination Survey
NRI: Net reclassification improvement
OR: Odds ratio
PF: Physical frailty
ROC: Recevier operating charaxteristic
SD: Standard deviation
TICS-10: The Telephone Interview of Cognitive Status-10

## References

[1] Ferrucci L, Levine ME, Kuo PL, Simonsick EM (2018). Time and the metrics of aging. Circ Res.

[2] Robertson DA, Savva GM, Kenny RA (2013). Frailty and cognitive impairment--a review of the evidence and causal mechanisms. Ageing Res Rev.

[3] Aliberti MJR, Cenzer IS, Smith AK, Lee SJ, Yaffe K, Covinsky KE (2019). Assessing risk for adverse outcomes in older adults: the need to include both physical frailty and cognition. J Am Geriatr Soc.

[4] Feng L, Zin Nyunt MS, Gao Q, Feng L, Yap KB, Ng TP (2017). Cognitive frailty and adverse health outcomes: findings from the Singapore longitudinal ageing studies (SLAS). J Am Med Dir Assoc.

[5] Solfrizzi V, Scafato E, Seripa D, Lozupone M, Imbimbo BP, D'Amato A (2017). Reversible cognitive frailty, dementia, and all-cause mortality. The Italian longitudinal study on aging. J Am Med Dir Assoc.

[6] Yu R, Morley JE, Kwok T, Leung J, Cheung O, Woo J (2018). The effects of combinations of cognitive impairment and pre-frailty on adverse outcomes from a prospective community-based cohort study of older Chinese people. Front Med (Lausanne).

[7] Solfrizzi V, Scafato E, Lozupone M, Seripa D, Giannini M, Sardone R (2017). Additive role of a potentially reversible cognitive frailty model and inflammatory state on the risk of disability: the Italian longitudinal study on aging. Am J Geriatr Psychiatry.

[8] St John PD, Tyas SL, Griffith LE, Menec V (2017). The cumulative effect of frailty and cognition on mortality - results of a prospective cohort study. Int Psychogeriatr.

[9] Esteban-Cornejo I, Cabanas-Sanchez V, Higueras-Fresnillo S, Ortega FB, Kramer AF, Rodriguez-Artalejo F (2019). Cognitive frailty and mortality in a National Cohort of older adults: the role of physical activity. Mayo Clin Proc.

[10] Ge ML, Carlson MC, Bandeen-Roche K, Chu NM, Tian J, Kasper JD (2020). U.S. national profile of older adults with cognitive impairment alone, physical frailty alone, and both. J Am Geriatr Soc.

[11] Chen C, Park J, Wu C, Xue Q, Agogo G, Han L (2020). Cognitive frailty in relation to adverse health outcomes independent of multimorbidity: results from the China health and retirement longitudinal study. Aging (Albany NY).

[12] Cano A (2015). Cognitive frailty, a new target for healthy ageing. Maturitas.

[13] Ruan Q, Yu Z, Chen M, Bao Z, Li J, He W (2015). Cognitive frailty, a novel target for the prevention of elderly dependency. Ageing Res Rev.

[14] Xue QL, Buta B, Ma L, Ge M, Carlson M (2019). Integrating frailty and cognitive phenotypes: why, how, now what?. Curr Geriatr Rep.

[15] Kelaiditi E, Cesari M, Canevelli M, van Kan GA, Ousset PJ, Gillette-Guyonnet S (2013). Cognitive frailty: rational and definition from an (I.a.N.a./I.a.G.G.) international consensus group. J Nutr Health Aging.

[16] Clegg A, Young J, Iliffe S, Rikkert MO, Rockwood K (2013). Frailty in elderly people. Lancet (London, England).

[17] Fried LP, Tangen CM, Walston J, Newman AB, Hirsch C, Gottdiener J (2001). Frailty in older adults: evidence for a phenotype. J Gerontol A Biol Sci Med Sci.

[18] Mitnitski AB, Mogilner AJ, Rockwood K (2001). Accumulation of deficits as a proxy measure of aging. Sci World J.

[19] Verghese J, Annweiler C, Ayers E, Barzilai N, Beauchet O, Bennett DA (2014). Motoric cognitive risk syndrome: multicountry prevalence and dementia risk. Neurology.

[20] Bortone I, Sardone R, Lampignano L, Castellana F, Zupo R, Lozupone M, et al. How gait influences frailty models and health-related outcomes in clinical-based and population-based studies: a systematic review. J Cachexia Sarcopenia Muscle. 2021. 10.1002/jcsm.12667. 10.1002/jcsm.12667 PMC8061366 33590975

[21] Wu C, Smit E, Xue QL, Odden MC (2017). Prevalence and correlates of frailty among community-dwelling Chinese older adults: the China health and retirement longitudinal study. J Gerontol A Biol Sci Med Sci.

[22] Varadaraj V, Lee MJ, Tian J, Ramulu PY, Bandeen-Roche K, Swenor BK (2019). Near vision impairment and frailty: evidence of an association. Am J Ophthalmol.

[23] Searle SD, Mitnitski A, Gahbauer EA, Gill TM, Rockwood K (2008). A standard procedure for creating a frailty index. BMC Geriatr.

[24] Hoover M, Rotermann M, Sanmartin C, Bernier J (2013). Validation of an index to estimate the prevalence of frailty among community-dwelling seniors. Health Rep.

[25] Verghese J, Wang C, Lipton RB, Holtzer R (2013). Motoric cognitive risk syndrome and the risk of dementia. J Gerontol A Biol Sci Med Sci.

[26] Sugimoto T, Sakurai T, Ono R, Kimura A, Saji N, Niida S (2018). Epidemiological and clinical significance of cognitive frailty: a mini review. Ageing Res Rev.

[27] Lim YJ, Ng YS, Sultana R, Tay EL, Mah SM, Chan CHN (2020). Frailty assessment in community-dwelling older adults a comparison of 3 diagnostic instruments. J Nutr Health Aging.

[28] Wang ZD, Yao S, Shi GP, Wang Y, Shi JM, Guo JH (2020). Frailty index is associated with increased risk of elevated BNP in an elderly population: the Rugao longevity and ageing study. Aging Clin Exp Res.

[29] Ruan Y, Guo Y, Kowal P, Lu Y, Liu C, Sun S (2019). Association between anemia and frailty in 13,175 community-dwelling adults aged 50 years and older in China. BMC Geriatr.

[30] Dugravot A, Fayosse A, Dumurgier J, Bouillon K, Rayana TB, Schnitzler A (2020). Social inequalities in multimorbidity, frailty, disability, and transitions to mortality: a 24-year follow-up of the Whitehall II cohort study. Lancet Public Health.

[31] Castellana F, Lampignano L, Bortone I, Zupo R, Lozupone M, Griseta C, et al. Physical frailty, multimorbidity, and all-cause mortality in an older population from southern Italy: results from the Salus in Apulia study. J Am Med Dir Assoc. 2021. 10.1016/j.jamda.2020.12.026. 10.1016/j.jamda.2020.12.026 33493467

[32] Won CW, Lee Y, Kim S, Yoo J, Kim M, Ng TP (2018). Modified criteria for diagnosing "cognitive frailty". Psychiatry Investig.

[33] Aguayo GA, Vaillant MT, Donneau AF, Schritz A, Stranges S, Malisoux L (2018). Comparative analysis of the association between 35 frailty scores and cardiovascular events, cancer, and total mortality in an elderly general population in England: an observational study. PLoS Med.

[34] Panza F, Lozupone M, Solfrizzi V, Sardone R, Dibello V, Di Lena L (2018). Different cognitive frailty models and health- and cognitive-related outcomes in older age: from epidemiology to prevention. J Alzheimers Dis.

[35] Vella Azzopardi R, Beyer I, Vermeiren S, Petrovic M, Van Den Noortgate N, Bautmans I (2018). Increasing use of cognitive measures in the operational definition of frailty-a systematic review. Ageing Res Rev.

[36] Blodgett JM, Theou O, Howlett SE, Rockwood K (2017). A frailty index from common clinical and laboratory tests predicts increased risk of death across the life course. Geroscience.

