# Supplementary material for: Aging metrics incorporating cognitive and physical function capture mortality risk: results from two prospective cohort studies
Source: BMC Geriatr. 2022 Apr 28;22:378. doi: 10.1186/s12877-022-02913-y (PMC9052591; doi:10.1186/s12877-022-02913-y)
Supplement: Supplementary file 1 — Additional file 1: Figure S1. Roadmap for the comprehensive analyses of the three aging metrics incorporating cognitive and physical function. [file 12877_2022_2913_MOESM1_ESM.docx]

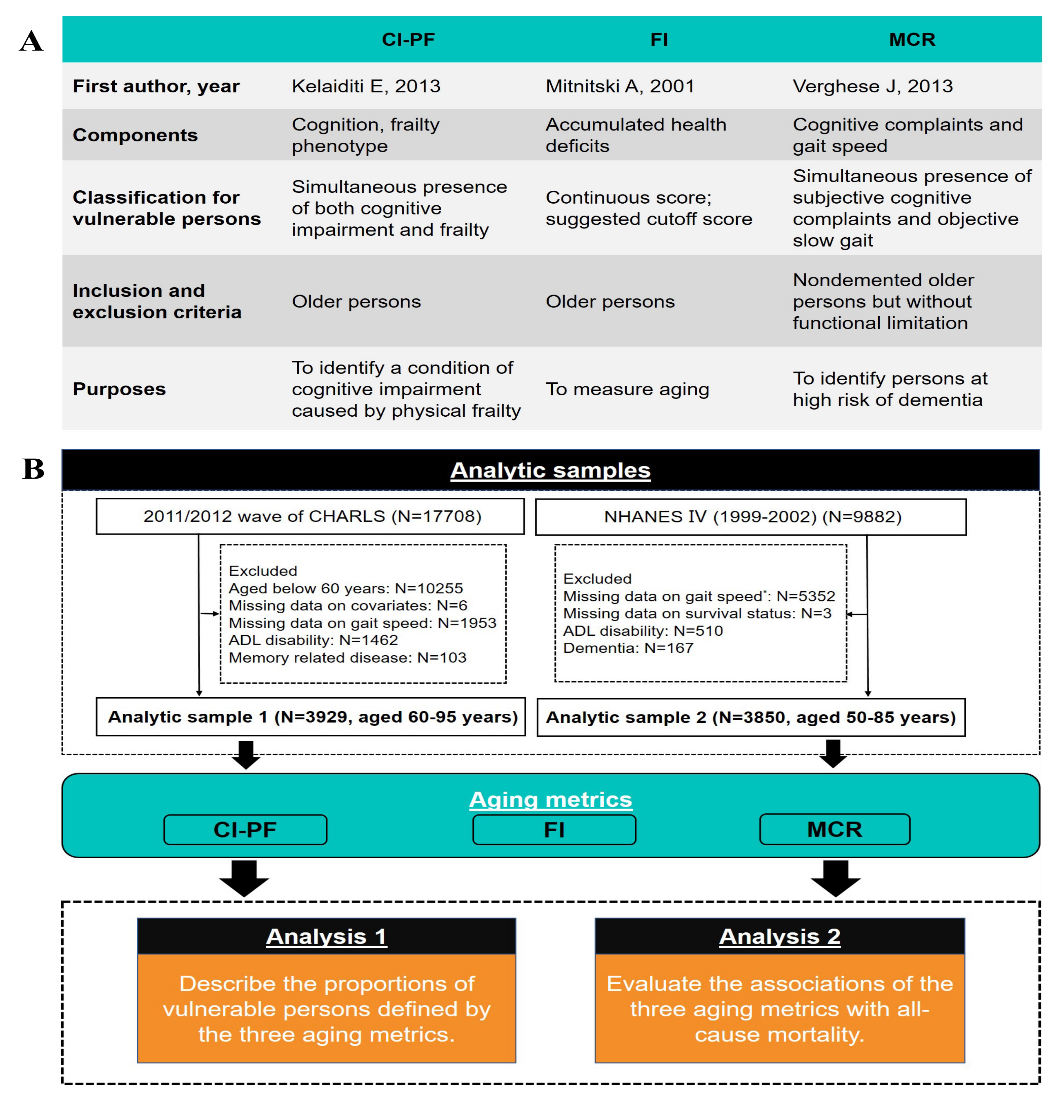


**Figure S1. Roadmap for the comprehensive analyses of the three aging metrics incorporating cognitive and physical function.** CHARLS, China Health and Retirement Longitudinal Study; NHANES, National Health and Nutrition Examination Survey; CI-PF, cognitive impairment and physical frailty; FI, frailty index; MCR, Motoric Cognitive Risk syndrome. A describes the details on the three aging metrics incorporating cognitive and physical function. B shows the assembly of analytic samples and the detailed analyses performed in this study. ^*^ The walk testing was only measured in participants who aged 50 years and older.
