# Supplementary material for: Aging metrics incorporating cognitive and physical function capture mortality risk: results from two prospective cohort studies
Source: BMC Geriatr. 2022 Apr 28;22:378. doi: 10.1186/s12877-022-02913-y (PMC9052591; doi:10.1186/s12877-022-02913-y)
Supplement: Supplementary file 2 — Additional file 2. Detailed description of the Methods. [file 12877_2022_2913_MOESM2_ESM.docx]

**Detailed description of the Methods.**

**Study population**

The China Health and Retirement Longitudinal Study (CHARLS) targeted Chinese community-dwelling persons aged 45 years and older and their spouses. The CHARLS used a multistage sampling strategy covering 28 provinces, 150 counties/districts, and 450 villages/urban communities across the country. Persons were first recruited in 2011/2012, and completed three follow-up visits biennially up to 2017/2018. Details of the CHARLS survey have been described in previous studies [1]. The CHARLS study offers a wide range of information on socioeconomic status and health. The CHARLS was approved by the Biomedical Ethics Review Committee of Peking University, and all persons provided informed consent. The CHARLS data are publicly available through the CHARLS website (<http://charls.pku.edu.cn/en>). out of 17708 persons aged 45 years and older enrolled in the baseline survey (2011/2012), we excluded those aged below 60 years (N=10255; because gait speed was measured only in older persons aged 60 years and over), with missing data on covariates (N=6), missing data on gait speed (N=1953), who had disability in activities of daily living (ADL) (N=1462), or had the memory-related disease (e.g., Alzheimer's disease, brain atrophy, and Parkinson's disease) (N=103), leaving the analytic sample 1 of 3929 persons aged 60-95 years (Figure S1B in the **Additional file 1**).

Since 1999, the US National Health and Nutrition Examination Survey (NHANES) was designed as a continuous, annual survey, comprised of a nationally representative sample of the US population. Every year, approximately 5000 persons among the 7000 randomly selected individuals could complete both the comprehensive health screening and questionnaire interview. The survey data are publicly released in 2-year cycles after 1999. The response rates were 76% (N=9282) and 80% (N=10477) in the 1999-2000 and the 2001-2002 cycles, respectively [2]. The NHANES was approved by the National Center for Health Statistics Research Ethics Review Board, and all persons provided informed consent. Detailed questionnaire instrumentals, laboratory methods, procedure manuals, brochures, and consent documents of the NHANES 1999-2002 are available through the NHANES website (<https://www.cdc.gov/nchs/nhanes/index.htm>) [3]. Out of the 9882 persons aged 20 years and older, we excluded persons with missing data on gait speed (N=5352; gait speed measurement is needed to construct both PF and MCR, but from 2003-2004 cycle on, gait speed was no longer captured in NHANES) or final mortality status (N=3), who had disability in ADL (N=510), or had dementia (N=167), leaving the analytic sample 2 of 3850 persons aged 50-85 years (Figure S1B in the **Additional file 1**).

**Measures**

**Cognitive function**

In the CHARLS, cognitive function was assessed by three tests, including the Telephone Interview of Cognitive Status-10 (TICS-10), word recall, and figure drawing. Ten items from TICS-10 included date (month, day, and year), day of the week, the season of the year, and serial subtraction of 7 from 100 (up to five times). Summing the number of correct answers to these questions resulted in a TICS-10 score, ranging from 0 to 10. For word recall, persons were asked to immediately recall as many words as they could in any order immediately after interviewers read 10 Chinese nouns (i.e., immediate recall). Four to ten minutes later, persons were asked to recall as many of the original words as possible (i.e., delayed recall) [4, 5]. The episodic memory was assessed by the average number of immediate and delayed word recalls, ranging from 0 to 10 [6]. The visuospatial ability was assessed by figure drawing. Persons were shown a picture of two overlapped pentagons and asked to draw a similar figure [7]. Persons who completed the task received a score of 1, and those who failed received a score of 0. The summary score ranged from 0 to 21, with higher scores indicating better cognitive function [4, 8]. According to the literature [9], persons were classified as having cognitive impairment if their summary score fell more than 1 standard deviation (SD) below age-appropriate norms; otherwise, they were classified as having normal cognition.

During the 1999-2002 cycle, the NHANES used the Digit Symbol Substitution Test (DSST) to assess cognitive function. The DSST is widely used as a sensitive measure of frontal lobe executive functions [10]. After signing a licensing agreement with the Psychological Corporation, in this coding exercise, persons were asked to copy the corresponding symbols in the 133 boxes that are paired with the numbers in 120 seconds. The summary score of the DSST ranged from 0 to 133, with higher scores indicating better cognitive function. According to the literature [11], persons were classified as having cognitive impairment if their summary score was below the median DSST score (i.e., 40); otherwise, they were classified as having normal cognition.

**Physical frailty (PF)**

The PF was measured using the Fried frailty phenotype approach [12], and had been previously developed and validated in the CHARLS [13] and NHANES [14]. Persons were classified as frail if they met ≥3 of the five items; otherwise, they were classified as non-frail.

Five items in CHARLS are as follows:

1. Shrinking was established as self-reported loss of 5 or more kg in the previous year or having a body mass index (BMI) of 18.5 kg/m^2^ or less.

2. Weakness was defined as the lowest quintile of maximum handgrip strength (either hand; two trials for each; measured in a standing position with the arm bent at 90 degrees using the dynamometer (WCS-100, Nantong, China)), adjusting for sex and BMI.

3. Exhaustion was determined according to two questions from the Center for Epidemiological Studies-Depression scale: “I could not get going” and “I felt everything I did was an effort”.

4. Slowness was defined as the lowest quintile on the average of two-timed walk tests over a 2.5-meter course, at usual pace, adjusting for sex and standing height.

5. Inactivity was determined if persons self-reported that they did not walk 10 or more minutes continuously during a usual week.

Five items in NHANES are as follows:

1. Shrinking was established as self-reported ≥10 lb or ≥5% unintentional weight loss in the previous year or having a BMI of 18.5 kg/m^2^ or less.

2. Weakness was defined as if persons answered ‘‘some difficulty,’’ “much difficulty,’’ or ‘‘unable to do’’ when they were asked how much difficulty they have lifting or carrying something as heavy as 10 pounds, such as a sack of potatoes or rice.

3. Exhaustion was defined as if persons responded "some difficulty’’ or ‘‘much difficulty’’ when they were asked how much difficulty they have walking from 1 room to another on the same level by themselves without using special equipment.

4. Slowness was defined as the lowest quintile time of completing a 20-foot walk, at usual pace, among the population, adjusting for sex and standing height.

5. Inactivity was determined if persons self-reported ‘‘less active’’ when they were asked how active they were as compared to most males/females at their age.

**Functional metrics incorporating cognitive and physical function**

We considered three existing measures: the combined presence of cognitive impairment and PF (referred to as CI-PF), frailty index (FI), and Motoric Cognitive Risk syndrome (MCR) in this study.

**Version 1—CI-PF**

The CI-PF was defined as the simultaneous presence of both cognitive impairment and PF in non-demented older persons, proposed in 2013 by the International Association of Gerontology and Geriatrics (IAGG) consensus group [15]. Based on the two components, i.e., cognitive impairment and PF, we defined four combined groups as done in previous studies [16, 17]: normal cognition & non-frailty, cognitive impairment & non-frailty, normal cognition & frailty, and cognitive impairment & frailty. The cognitive impairment and frailty group was defined as vulnerable.

**Version 2—FI**

The FI was based on the degree of accumulation of health deficits and represents an alternative instrument of frailty that incorporates more health dimensions, including comorbidities, cognitive function, psychological factors, symptoms, and disabilities [18]. FI score was calculated as a ratio of counting the number of deficits in a person out of the total possible deficits considered [19], ranges from 0 to 1. For example, a person with 9 of a total possible of 36 deficits would have an FI score of 9/36=0.25, in NHANES. We constructed 39-item and 36-item version of self-report FI in CHARLS and NHANES, respectively. Any person who had missing data on 20% or less of the variables were retained and the missing values were imputed with the median.

To compare with the other two versions of cognitive frailty, we categorized FI into three groups, based on widely used cut-off values [20]. A FI ≤0.10 was considered as non-frail, 0.10< FI ≤0.21 was pre-frail, and FI >0.21 was frail. The group with frailty was defined as vulnerable.

**Thirty-nine items in CHARLS are as follows:**

Item 1: Has hypertension.

Item 2: Has dyslipidemia.

Item 3: Has diabetes or high blood glucose.

Item 4: Has chronic lung diseases.

Item 5: Has liver disease.

Item 6: Has heart disease.

Item 7: Has stroke.

Item 8: Has kidney disease.

Item 9: Has stomach or other digestive diseases.

Item 10: Has emotional, nervous, or psychiatric problems.

Item 11: Has memory-related disease.

Item 12: Has arthritis or rheumatism.

Item 13: Has asthma.

Item 14: Physical disabilities.

Item 15: Brain damage/mental retardation.

Item 16: Permanent vision damage.

Item 17: Permanent hearing damage.

Item 18: Self-rating of health.

Item 19: Limitations in dressing.

Item 20: Limitations in eating.

Item 21: Limitations in bathing or showering.

Item 22: Limitations in getting into or out of bed.

Item 23: Limitations in using the toilet.

Item 24: Difficulty with controlling urination and defecation.

Item 25: Limitations in doing household chores.

Item 26: Limitations in preparing hot meals.

Item 27: Limitations in shopping.

Item 28: Limitations in managing money.

Item 29: Limitations in taking medications.

Item 30: Limitations in running/jogging 1 kilometer.

Item 31: Limitations in walking 1 kilometer.

Item 32: Limitations in climbing several flights of stairs.

Item 33: Limitations in getting up from a chair after sitting for a long period.

Item 34: Limitations in crouching, kneeling, or stooping.

Item 35: Limitations in reaching or extending arms.

Item 36: Limitations in lifting weights over 5 kilograms.

Item 37: Limitations in picking up a coin from a table.

Item 38: Has depressive symptom (the score of 10-item Center for Epidemiologic Studies Depression Scale (CESD-10) ≥10).

Item 39: Cognitive impairment (1-(the cognition score/21)).

**Thirty-six items in NHANES are as follows:**

Item 1: General hearing.

Item 2: Has high blood pressure.

Item 3: Self-reported poor health.

Item 4: Has diabetes.

Item 5: Frequency of healthcare use.

Item 6: Health compared to 1 year ago.

Item 7: Overnight hospital stays.

Item 8: Leaked/lost control of urine.

Item 9: Weak/failing kidneys.

Item 10: Has angina/angina pectoris.

Item 11: Has cancer.

Item 12: Has arthritis.

Item 13: Has heart attack.

Item 14: Has heart disease.

Item 15: Has thyroid condition.

Item 16: Has stroke.

Item 17: Has broken hip.

Item 18: Has osteoporosis.

Item 19: Confusion or inability to remember things.

Item 20: Difficulty in attending social events.

Item 21: Difficulty in dressing yourself difficulty.

Item 22: Difficulty in getting in and out of bed.

Item 23: Difficulty in grasping/holding small objects.

Item 24: Difficulty in lifting or carrying.

Item 25: Difficulty in managing money.

Item 26: Difficulty in preparing meals.

Item 27: Difficulty in pushing or pulling large objects.

Item 28: Difficulty in standing up from the armless chair.

Item 29: Difficulty in stooping, crouching, kneeling.

Item 30: Difficulty in using fork and knife.

Item 31: Difficulty in walking between rooms on the same floor.

Item 32: Have you taken or used any prescription medicines in the past month?

Item 33: Cough regularly.

Item 34: General vision.

Item 35: Cataract operation.

Item 36: Difficulty in seeing steps/curbs in dim light.

**Version 3—MCR**

The MCR was defined as the simultaneous presence of both subjective cognitive complaints and objective slow gait, in the absence of a diagnosis of dementia and difficulty in ADL [21]. The group of persons with MCR was defined as vulnerable.

The detailed four items are as follows:

1. Cognitive complaint was determined if persons self-reported poor memory (CHARLS), or answered ‘‘yes’’ when they were asked if they suffered the memory impairment problem (NHANES).

2. Slow gait was defined as 1 SD or more below age- and sex-appropriate norms of the time to complete a 2.5-meter (CHARLS) or 20-foot (NHANES) walk.

3. Preserved function of ADL was evaluated on a standardized scale of basic activities of daily living (BADL), which included 5 items (dressing, bathing, eating, getting in/out of bed, and using the toilet). For each item, persons were asked, “Do you have difficulty in performing the task?” Those persons who need personal assistance in performing one or more of the corresponding activities were classified as having BADL disability.

4. Absence of dementia. Due to the unavailable information of dementia diagnosis in CHARLS, we used a modified definition, in which persons were classified as having dementia if they reported that they have a memory-related disease (e.g., Alzheimer's disease, brain atrophy, and Parkinson's disease); otherwise, they were defined as without dementia. Similarly, in NHANES, we used a modified definition, in which persons were classified as having dementia if their summary score below the lowest decile of the DSST score (i.e., 14); otherwise, they were defined as without dementia [22].

**References:**

1. Zhao Y, Hu Y, Smith JP, Strauss J, Yang G. Cohort profile: the China Health and Retirement Longitudinal Study (CHARLS). Int J Epidemiol. 2014;43(1):61-68. doi:10.1093/ije/dys203

2. Curtin LR, Mohadjer LK, Dohrmann SM, Montaquila JM, Kruszan-Moran D, Mirel LB*, et al*. The National Health and Nutrition Examination Survey: Sample Design, 1999-2006. Vital Health Stat 2. 2012(155):1-39.

3. Statistics. NCfH. National Health and Nutrition Examination Survey. Atlanta: Centers for Disease Control and Prevention. 2018 [cited 2018 Nov 29].

4. Huang W, Zhou Y. Effects of education on cognition at older ages: evidence from China's Great Famine. Soc Sci Med. 2013;98:54-62. doi:10.1016/j.socscimed.2013.08.021

5. Lei X, Smith JP, Sun X, Zhao Y. Gender Differences in Cognition in China and Reasons for Change over Time: Evidence from CHARLS. J Econ Ageing. 2014;4:46-55. doi:10.1016/j.jeoa.2013.11.001

6. McArdle JJ, Fisher GG, Kadlec KM. Latent variable analyses of age trends of cognition in the Health and Retirement Study, 1992-2004. Psychol Aging. 2007;22(3):525-545. doi:10.1037/0882-7974.22.3.525

7. Mathuranath PS, Nestor PJ, Berrios GE, Rakowicz W, Hodges JR. A brief cognitive test battery to differentiate Alzheimer's disease and frontotemporal dementia. Neurology. 2000;55(11):1613-1620. doi:10.1212/01.wnl.0000434309.85312.19

8. Li J, Cacchione PZ, Hodgson N, Riegel B, Keenan BT, Scharf MT*, et al*. Afternoon Napping and Cognition in Chinese Older Adults: Findings from the China Health and Retirement Longitudinal Study Baseline Assessment. J Am Geriatr Soc. 2017;65(2):373-380. doi:10.1111/jgs.14368

9. Jak AJ, Bondi MW, Delano-Wood L, Wierenga C, Corey-Bloom J, Salmon DP*, et al*. Quantification of five neuropsychological approaches to defining mild cognitive impairment. Am J Geriatr Psychiatry. 2009;17(5):368-375. doi:10.1097/JGP.0b013e31819431d5

10. Laurent A, Biloa-Tang M, Bougerol T, Duly D, Anchisi AM, Bosson JL*, et al*. Executive/attentional performance and measures of schizotypy in patients with schizophrenia and in their nonpsychotic first-degree relatives. Schizophr Res. 2000;46(2-3):269-283. doi:10.1016/s0920-9964(99)00232-7

11. Loprinzi PD, Crush E, Joyner C. Cardiovascular disease biomarkers on cognitive function in older adults: Joint effects of cardiovascular disease biomarkers and cognitive function on mortality risk. Prev Med. 2017;94:27-30. doi:10.1016/j.ypmed.2016.11.011

12. Fried LP, Tangen CM, Walston J, Newman AB, Hirsch C, Gottdiener J*, et al*. Frailty in older adults: evidence for a phenotype. J Gerontol A Biol Sci Med Sci. 2001;56(3):M146-156. doi:10.1093/gerona/56.3.m146

13. Wu C, Smit E, Xue QL, Odden MC. Prevalence and Correlates of Frailty Among Community-Dwelling Chinese Older Adults: The China Health and Retirement Longitudinal Study. J Gerontol A Biol Sci Med Sci. 2017;73(1):102-108. doi:10.1093/gerona/glx098

14. Varadaraj V, Lee MJ, Tian J, Ramulu PY, Bandeen-Roche K, Swenor BK. Near Vision Impairment and Frailty: Evidence of an Association. Am J Ophthalmol. 2019;208:234-241. doi:10.1016/j.ajo.2019.08.009

15. Kelaiditi E, Cesari M, Canevelli M, van Kan GA, Ousset PJ, Gillette-Guyonnet S*, et al*. Cognitive frailty: rational and definition from an (I.A.N.A./I.A.G.G.) international consensus group. J Nutr Health Aging. 2013;17(9):726-734. doi:10.1007/s12603-013-0367-2

16. Chen C, Park J, Wu C, Xue Q, Agogo G, Han L*, et al*. Cognitive frailty in relation to adverse health outcomes independent of multimorbidity: results from the China health and retirement longitudinal study. Aging (Albany NY) 2020;12(22):23129-23145. doi:10.18632/aging.104078

17. Kelaiditi E, Cesari M, Canevelli M, van Kan GA, Ousset PJ, Gillette-Guyonnet S*, et al*. Cognitive frailty: Rational and definition from an (I.A.N.A./I.A.G.G.) International Consensus Group. J Nutr Health Aging. 2013;17(9):726-734. doi:10.1007/s12603-013-0367-2

18. Mitnitski AB, Mogilner AJ, Rockwood K. Accumulation of deficits as a proxy measure of aging. ScientificWorldJournal. 2001;1:323-336. doi:10.1100/tsw.2001.58

19. Searle SD, Mitnitski A, Gahbauer EA, Gill TM, Rockwood K. A standard procedure for creating a frailty index. BMC Geriatr. 2008;8:24. doi:10.1186/1471-2318-8-24

20. Hoover M, Rotermann M, Sanmartin C, Bernier J. Validation of an index to estimate the prevalence of frailty among community-dwelling seniors. Health Rep. 2013;24(9):10-17.

21. Verghese J, Wang C, Lipton RB, Holtzer R. Motoric cognitive risk syndrome and the risk of dementia. J Gerontol A Biol Sci Med Sci. 2013;68(4):412-418. doi:10.1093/gerona/gls191

22. Chen SP, Bhattacharya J, Pershing S. Association of Vision Loss With Cognition in Older Adults. JAMA Ophthalmol. 2017;135(9):963-970. doi:10.1001/jamaophthalmol.2017.2838
