# Supplementary material for: Aging metrics incorporating cognitive and physical function capture mortality risk: results from two prospective cohort studies
Source: BMC Geriatr. 2022 Apr 28;22:378. doi: 10.1186/s12877-022-02913-y (PMC9052591; doi:10.1186/s12877-022-02913-y)
Supplement: Supplementary file 3 — Additional file 3: Table S1A. Summary characteristics of the cognition status among different age groups, CHARLS 2011/2012 and NHANES 1999–2002. Table S1B. Characteristics of the included and excluded population. Table S1C. Distribution of the participants with CI-PF and MCR across the FI groups, CHARLS 2011/2012 and NHANES 1999–2002. [file 12877_2022_2913_MOESM3_ESM.docx]

**Table S1A. Summary characteristics of the cognition status among different age groups, CHARLS 2011/2012 and NHANES 1999–2002.**

| **Characteristics** | **CHARLS** | | | | |  | **NHANES** | | | |
| --- | --- | --- | --- | --- | --- | --- | --- | --- | --- | --- |
|  | Total | **Age groups, years** | | | |  | Total | **Age groups, years** | | |
|  |  | 60-69 | | 70-79 | 80-95 |  |  | 50-59 | 60-69 | 70-85 |
| N | 3929 | | 2694 | 1044 | 191 |  | 3850 | 1099 | 1348 | 1403 |
| The cognition score ^a^, mean ± SD | 9.8 ± 4.3 | | 10.52 ± 4.13 | 8.48 ± 4.32 | 6.64 ± 4.11 |  | 42.0 ± 14.7 | 41.0 ± 0 | 45.45 ± 18.32 | 39.41 ± 15.85 |
| Cognitive impairment, % | 1348 (34.3) | | 874 (32.4) | 399 (38.2) | 75 (39.3) |  | 1055 (27.4) | 0 (0) | 432 (32.1) | 623 (44.4) |

CHARLS, China Health and Retirement Longitudinal Study; NHANES, National Health and Nutrition Examination Survey; SD, standard deviation.

Notes: ^a^ In CHARLS, cognitive function was assessed by three tests, including the Telephone Interview of Cognitive Status-10 (TICS-10), word recall, and figure drawing. In NHANES, cognitive function was assessed by the Digit Symbol Substitution Test.

**Table S1B. Characteristics of the included and excluded population ^a^.**

| **Characteristics** | **CHARLS** | | |  | **NHANES** | | |
| --- | --- | --- | --- | --- | --- | --- | --- |
|  | **Excluded** | **Included** | ***P* value** |  | **Excluded** | **Included** | ***P* value** |
| N | 13779 | 3929 |  |  | 6032 | 3850 | ─ |
| Age, mean ± SD | 56.2 ± 9.7 | 67.4 ± 6.3 | <0.001 |  | 38.1 ± 14.2 | 65.6 ± 9.6 | <0.001 |
| Male, % | 6374 (46.3) | 2102 (53.5) | <0.001 |  | 2710 (44.9) | 1927 (50.1) | <0.001 |
| Residence, rural, % | 8110 (58.9) | 2427 (61.8) | 0.001 |  | ─ | ─ |  |
| Race/Ethnicity ^a^, % |  |  |  |  |  |  | <0.001 |
| Non-Hispanic white | ─ | ─ |  |  | 2666 (45.7) | 2130 (56.6) |  |
| Non-Hispanic black | ─ | ─ |  |  | 1233 (21.1) | 673 (17.9) |  |
| Hispanic | ─ | ─ |  |  | 1937 (33.2) | 957 (25.5) |  |
| Education ^b^, % |  |  | <0.001 |  |  |  | <0.001 |
| Category 1 | 3555 (25.8) | 1296 (33.0) |  |  | 1869 (31.1) | 1496 (39.0) |  |
| Category 2 | 5061 (36.8) | 1859 (47.3) |  |  | 1411 (61.9) | 868 (22.6) |  |
| Category 3 | 3166 (23.0) | 511 (13.0) |  |  | 1584 (26.4) | 799 (20.8) |  |
| Category 4 | 1672 (12.2) | 195 (5.0) |  |  | 1145 (19.1) | 677 (17.6) |  |
| Category 5 | 308 (2.2) | 68 (1.7) |  |  | ─ | ─ |  |
| Disease counts ^c^, % |  |  | <0.001 |  |  |  | <0.001 |
| 0 | 4685 (34.3) | 1116 (28.4) |  |  | 4060 (67.3) | 805 (20.9) |  |
| 1 | 4234 (31.0) | 1252 (31.9) |  |  | 1245 (20.6) | 1138 (29.6) |  |
| 2 | 2704 (19.8) | 885 (22.5) |  |  | 415 (30.2) | 961 (25.0) |  |
| 3 | 1221 (9.0) | 426 (10.8) |  |  | 187 (24.6) | 574 (14.9) |  |
| ≥4 | 804 (5.9) | 250 (6.4) |  |  | 125 (25.2) | 372 (9.7) |  |

CHARLS, China Health and Retirement Longitudinal Study; NHANES, National Health and Nutrition Examination Survey; SD, standard deviation.

Notes: ^a^ There were missing data on sex (CHARLS: N=2; NHANES: N=409), race/ethnicity (NHANES: N=695), education (CHARLS: N=17; NHANES: N=442), and disease count (CHARLS: N=131; NHANES: N=409).

^b^ In CHARLS, category 1 to 5 indicates “illiteracy”, “elementary”, “middle”, “senior” and “college and higher than college”, respectively; In NHANES, category 1 to 4 indicates “lower than high school”, “high school or general educational development”, “some college”, and “college”, respectively.

^c^ In CHARLS, chronic diseases included hypertension, diabetes or high blood sugar, cancer or malignant tumor, chronic lung disease, heart problems, stroke, kidney disease, stomach or other digestive diseases, arthritis or rheumatism, and asthma. In NHANES, chronic diseases included congestive heart failure, stroke, cancer, chronic bronchitis, emphysema, cataracts, arthritis, type 2 diabetes, hypertension, and myocardial infarction.

**Table S1C. Distribution of the participants with CI-PF and MCR across the FI groups, CHARLS 2011/2012 and NHANES 1999-2002.**

| **Aging metrics** | **CHARLS** | | | | |  | **NHANES** | | | |
| --- | --- | --- | --- | --- | --- | --- | --- | --- | --- | --- |
|  | **FI, %** | | | **Total** | |  | **FI, %** | | | **Total** |
|  | **Non-frail** | **Pre-frail** | **Frail** | |  |  | **Non-frail** | **Pre-frail** | **Frail** |  |
| **CI-PF** |  |  |  | |  |  |  |  |  |  |
| Normal cognition & non-frailty, % | 1334 (72.2) | 854 (59.8) | 313 (47.9) | | 2501 (63.7) |  | 970 (82.5) | 1174 (71.6) | 417 (40.3) | 2561 (66.5) |
| Cognitive impairment & non-frailty, % | 484 (26.2) | 517 (36.2) | 262 (40.1) | | 1263 (32.2) |  | 205 (17.4) | 430 (26.2) | 316 (30.5) | 951 (24.7) |
| Normal cognition & frailty, % | 16 (0.9) | 31 (2.2) | 33 (5.1) | | 80 (2.0) |  | 1 (0.1) | 26 (1.6) | 207 (20.0) | 234 (6.1) |
| Cognitive impairment & frailty, % | 14 (0.7) | 26 (1.8) | 45 (6.9) | | 85 (2.2) |  | 0 (0) | 9 (0.6) | 95 (9.2) | 104 (2.7) |
| **MCR** |  |  |  | |  |  |  |  |  |  |
| Absence, % | 1652 (89.4) | 1095 (76.7) | 412 (63.1) | | 3159 (80.4) |  | 1176 (100) | 1605 (97.9) | 902 (87.1) | 3683 (95.7) |
| Presence, % | 196 (10.6) | 333 (23.3) | 241 (36.9) | | 770 (19.6) |  | 0 (0) | 34 (2.1) | 133 (12.9) | 167 (4.3) |
| **Total** | 1848 (47.0) | 1428 (36.4) | 653 (16.6) | | 3929 (100) |  | 1176 (30.6) | 1639(42.6) | 1035(26.9) | 3850 (100) |

CI-PF, cognitive impairment and physical frailty; MCR, Motoric Cognitive Risk syndrome; FI, frailty index; CHARLS, China Health and Retirement Longitudinal Study; NHANES, National Health and Nutrition Examination Survey.
