# Supplementary material for: Aging metrics incorporating cognitive and physical function capture mortality risk: results from two prospective cohort studies
Source: BMC Geriatr. 2022 Apr 28;22:378. doi: 10.1186/s12877-022-02913-y (PMC9052591; doi:10.1186/s12877-022-02913-y)
Supplement: Supplementary file 4 — Additional file 4: Table S3. Associations of the three aging metrics incorporating cognitive and physical function with all-cause mortality when additionally adjusting for comorbidities. Figure S2. Kaplan-Meier survival curves of the groups defined by the three aging metrics in NHANES. [file 12877_2022_2913_MOESM4_ESM.docx]

**[Table S2.](#bookmark68) Associations of the three aging metrics incorporating cognitive and physical function with all-cause mortality when additionally adjusting for number of comorbidities.**

| **Aging metrics** | | **Model 3** ^a^ |
| --- | --- | --- |
| **CHARLS** | | **OR (95% CI)** |
| **CI-PF** ^b^ | Normal cognition & non-frailty | Ref |
|  | Cognitive impairment & non-frailty | 1.35 (1.08, 1.69) |
|  | Normal cognition & frailty | 1.63 (0.95, 2.79) |
|  | Cognitive impairment & frailty | 2.41 (1.45, 4.01) |
| **FI** | Non-frail | Ref |
|  | Pre-frail | 1.30 (1.03, 1.65) |
|  | Frail | 1.92 (1.41, 2.63) |
| **MCR** | Absence | Ref |
|  | Presence | 1.13 (0.89, 1.45) |
|  |  |  |
| **NHANES** | | **HR (95% CI)** |
| **CI-PF** | Normal cognition & non-frailty | Ref |
|  | Cognitive impairment & non-frailty | 1.29 (1.14, 1.45) |
|  | Normal cognition & frailty | 2.51 (2.09, 3.02) |
|  | Cognitive impairment & frailty | 2.14 (1.67, 2.74) |
| **FI** | Non-frail |  |
|  | Pre-frail | 1.21 (1.04, 1.41) |
|  | Frail | 2.02 (1.68, 2.42) |
| **MCR** | Absence |  |
|  | Presence | 1.55 (1.28, 1.88) |

CHARLS, China Health and Retirement Longitudinal Study; OR, odds ratio; CI, confidence interval; CI-PF, cognitive impairment and physical frailty; FI, frailty index; MCR, Motoric Cognitive Risk syndrome; NHANES, National Health and Nutrition Examination Survey; HR, hazard ratio.

Notes: ^a^Model 3: adjusted for age, sex, education, residence (in CHARLS) or ethnicity/race (in NHANES), and number of comorbidities.

^b^As previously reported (Chen et al., 2020), significant differences in all-cause mortality between the four CI-PF groups were observed.
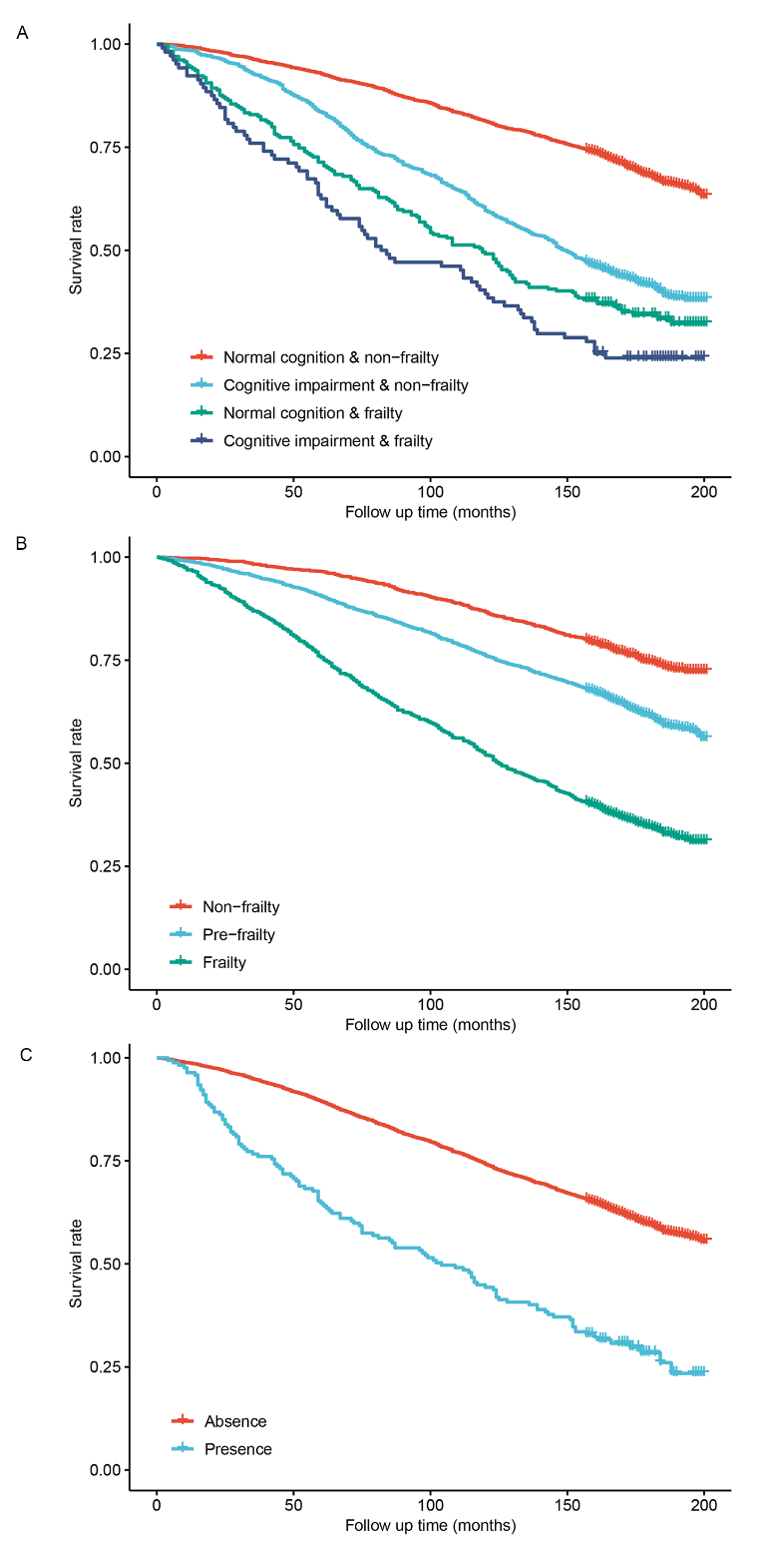
**Figure S2. Kaplan-Meier survival curves of the groups defined by the three aging metrics in NHANES.**

NHANES, National Health and Nutrition Examination Survey. The time-dependent risk of all-cause death by groups. The y-axis indicates the cumulative all-cause death probability, and the x-axis indicates follow-up time (in months). A, B and C show the Kaplan-Meier curves of the groups defined by the combined presence of cognitive impairment and physical frailty (CI-PF), frailty index (FI) and Motoric Cognitive Risk syndrome (MCR), respectively.
