# Supplementary material for: Aging metrics incorporating cognitive and physical function capture mortality risk: results from two prospective cohort studies
Source: BMC Geriatr. 2022 Apr 28;22:378. doi: 10.1186/s12877-022-02913-y (PMC9052591; doi:10.1186/s12877-022-02913-y)
Supplement: Supplementary file 5 — Additional file 5: Sensitivity Analyses Results. Table S4. Associations of the three aging metrics incorporating cognitive and physical function with all-cause mortality among older adults aged 60 years and over in NHANES (N = 2751). Figure S3. Association of the three aging metrics with all-cause mortality among older adults aged 60 years and over in NHANES (N = 2751). [file 12877_2022_2913_MOESM5_ESM.docx]

**Sensitivity Analyses Results**

We performed sensitivity analyses in which we excluded persons aged below 60 years (N=1099) in NHANES and found that the results remain unchanged (**Table S3** and **Figure S3**). When using the CI-PF, compared with the normal cognition & non-frailty group, the multivariable-adjusted HRs of the cognitive impairment & non-frailty group, normal cognition & frailty group, and cognitive impairment & frailty group for all-cause mortality were 1.45 (95% CI 1.28-1.65), 3.29 (95% CI 2.62-4.14), and 2.94 (95% CI 2.20-3.95), respectively. When using the FI, compared with the non-frail group, the multivariable-adjusted HRs of the pre-frail group and the frail group were 1.35 (95% CI 1.16-1.57), and 2.51 (95% CI 12.15-2.94), respectively. When using the MCR, compared with persons without MCR, the multivariable-adjusted HR of persons with MCR were 1.80 (95% CI 1.42-2.27). Compared with the basic model (with age and sex only), the models including CI-PF, FI, or MCR had better discrimination ability, as demonstrated by significantly increased C-statistics (range: 0.004 to 0.038). The performance of CI-PF, FI, and MCR was further demonstrated by significant improvements in reclassification as assessed by IDI (range: 0.008-0.048) and continuous NRI (range: 0.123-0.443).

[**Table S3.**](#bookmark68) **Associations of** **the three aging metrics incorporating cognitive and physical function with all-cause mortality among older adults aged 60 years and over in NHANES (N=2751).**

| **Aging metrics** | | **Model 1** | **Model 2** |
| --- | --- | --- | --- |
|  |  | **HR (95% CI)** | **HR (95% CI)** |
| **CI-PF** | Normal cognition & non-frailty | Ref | Ref |
|  | Cognitive impairment & non-frailty | 1.43 (1.27, 1.60) | 1.45 (1.28, 1.65) |
|  | Normal cognition & frailty | 3.25 (2.58, 4.09) | 3.29 (2.62, 4.14) |
|  | Cognitive impairment & frailty | 2.94 (2.24, 3.860) | 2.94 (2.20, 3.95) |
| **FI** | Non-frail | Ref | Ref |
|  | Pre-frail | 1.37 (1.19, 1.60) | 1.35 (1.16, 1.57) |
|  | Frail | 2.59 (2.22, 3.02) | 2.51 (2.15, 2.94) |
| **MCR** | Absence | Ref | Ref |
|  | Presence | 1.86 (1.49, 2.32) | 1.80 (1.42, 2.27) |

NHANES, National Health and Nutrition Examination Survey; HR, hazard ratio; CI, confidence interval; CI-PF, cognitive impairment and physical frailty; FI, frailty index; MCR, Motoric Cognitive Risk syndrome.

Model 1: adjusted for age, and sex.

Model 2: adjusted for age, sex, education, and ethnicity/race.


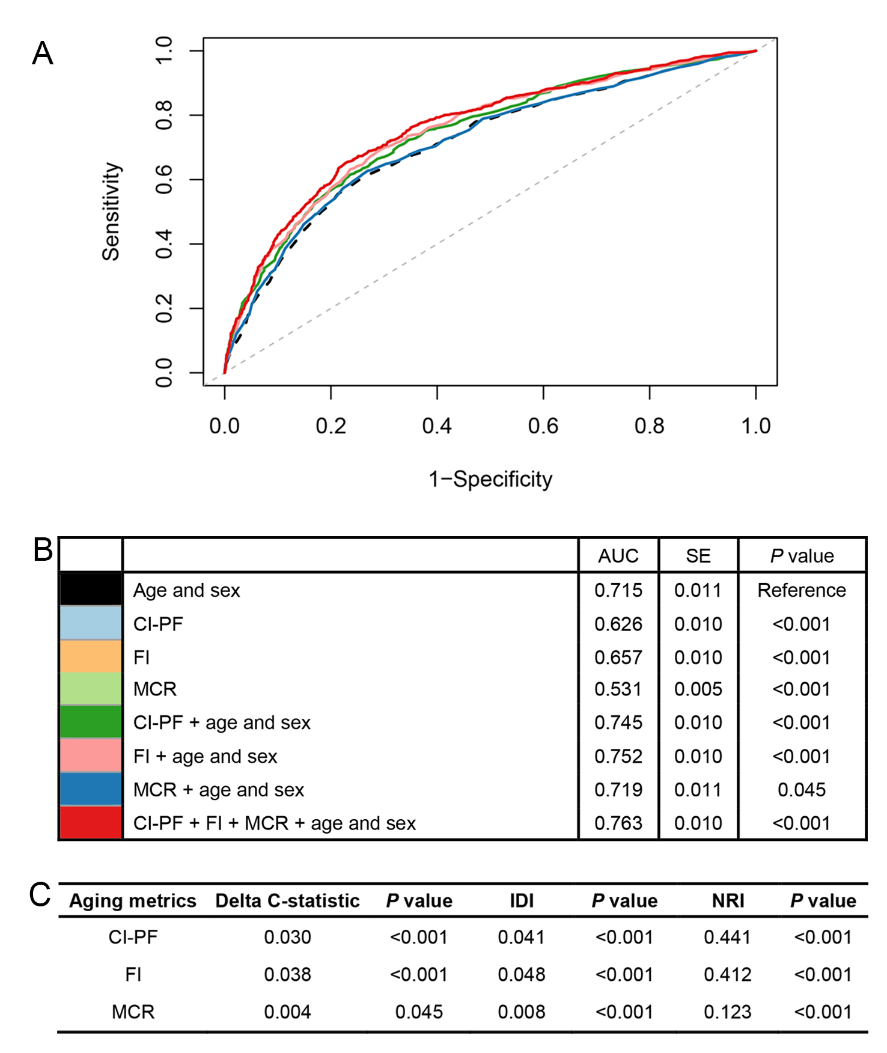
**Figure S3. Association of the three aging metrics with all-cause mortality among older adults aged 60 years and over in NHANES (N=2751).**

NHANES, National Health and Nutrition Examination Survey; CI-PF, cognitive impairment and physical frailty; FI, frailty index; MCR, Motoric Cognitive Risk syndrome; AUC, area under the curve; SE, standard error; IDI, integrated discrimination improvement; NRI, net reclassification index. We calculated the continuous NRI and IDI using R package “PredictABEL”, in comparison to that of the basic model with age and sex. NRI equals to x% means that compared with persons without outcome, persons with outcome were almost x% more likely to move up a category than down. IDI equals to x% means that the difference in average predicted risks between the persons with and without the outcome increased by x% in the updated model. A shows receiver-operator characteristics curves for prediction of all-cause mortality for three aging metrics. B shows the AUC for each model. C shows delta C-statistic, IDI, and NRI, in comparison to that of the basic model with age and sex.
